# The Effects of Reducing Sedentary Behavior Duration by Increasing Physical Activity, on Cognitive Function, Brain Function and Structure Across the Lifespan: A Systematic Review

**DOI:** 10.1002/alz.093104

**Published:** 2025-01-09

**Authors:** Natan Feter, Dominika Pindus

**Affiliations:** ^1^ Universidade Federal do Rio Grande do Sul, Porto Alegre Brazil; ^2^ University of Illinois Urbana‐Champaign, Champaign, IL USA

## Abstract

**Background:**

No study has synthesized evidence from randomized controlled trials (RCTs) testing the acute and chronic effects of reallocating sedentary time (ST) to physical activity (PA) on cognitive and brain health across the lifespan.

**Method:**

Seven academic databases were systematically searched on October 26, 2022, with an updated search on June 23, 2023. Inclusion criteria encompassed RCTs published in English involving healthy participants aged ≥ 4 years. Acute (lasting 3 hours to 13 days) and chronic (lasting ≥ 2 weeks) interventions aiming to reduce ST and/or prolonged ST by reallocating ST to PA to improve cognition, brain function, and structure were included. Two independent assessors evaluated the study quality using the Risk of Bias 2.0 tool.

**Result:**

We included 22 trials (n = 1,166) investigating acute (18 studies) and chronic (4 studies) effects on cognition (21 studies) and brain function (5 studies). No study investigated brain structure. Most studies included adults aged 18‐64 years (63.9%), followed by children and adolescents (4‐17 years) (27.3%) and older adults (≥65) (9.0%). Inconclusive evidence exists regarding the impact of reallocating ST to PA on brain function and cognition, attributed to the high risk of bias, limited number of studies, and diverse exercise protocols. However, emerging neurofunctional findings from a single chronic study suggest that relative to prolonged ST, introducing one bout of high‐intensity PA on most days enhanced neural efficiency of the hippocampus and parahippocampal gyrus during an episodic memory task in young adults. Interrupting ST with light PA bouts also acutely improved cerebrovascular function in young adults (1 out of 3 studies). The positive cognitive effects of acutely interrupting prolonged ST with PA were selective to inhibitory control (3 out of 6 studies) and mental flexibility (2 out of 5 studies), with largely null findings on other cognitive outcomes and chronic interventions.

**Conclusion:**

The evidence on the acute and chronic effects of reallocating ST to PA on brain function and cognition remains inconclusive with no studies measuring brain structure. However, specific studies suggest that reallocating ST to PA may chronically enhance hippocampal function and acutely improve inhibitory control, mental flexibility, and cerebrovascular function.

